# Connexin-43 channels are a pathway for discharging lactate from glycolytic pancreatic ductal adenocarcinoma cells

**DOI:** 10.1038/onc.2017.71

**Published:** 2017-04-03

**Authors:** T H Dovmark, M Saccomano, A Hulikova, F Alves, P Swietach

**Affiliations:** 1 Department of Physiology, Anatomy and Genetics, Oxford University, Oxford, UK; 2 Max-Planck-Institute of Experimental Medicine, Goettingen, Germany; 3 Institute for Diagnostic and Interventional Radiology, University Medical Center Goettingen, Goettingen, Germany; 4 Department of Haematology and Medical Oncology, University Medical Center Goettingen, Goettingen, Germany

## Abstract

Glycolytic cancer cells produce large quantities of lactate that must be removed to sustain metabolism in the absence of oxidative phosphorylation. The only venting mechanism described to do this at an adequate rate is H^+^-coupled lactate efflux on monocarboxylate transporters (MCTs). Outward MCT activity is, however, thermodynamically inhibited by extracellular acidity, a hallmark of solid tumours. This inhibition would feedback unfavourably on metabolism and growth, raising the possibility that other venting mechanisms become important in under-perfused tumours. We investigated connexin-assembled gap junctions as an alternative route for discharging lactate from pancreatic ductal adenocarcinoma (PDAC) cells. Diffusive coupling (calcein transmission) *in vitro* was strong between Colo357 cells, weaker yet hypoxia-inducible between BxPC3 cells, and very low between MiaPaCa2 cells. Coupling correlated with levels of connexin-43 (Cx43), a protein previously linked to late-stage disease. Evoked lactate dynamics, imaged in Colo357 spheroids using cytoplasmic pH as a read-out, indicated that lactate anions permeate gap junctions faster than highly-buffered H^+^ ions. At steady-state, junctional transmission of lactate (a chemical base) from the spheroid core had an alkalinizing effect on the rim, producing therein a milieu conducive for growth. Metabolite assays demonstrated that Cx43 knockdown increased cytoplasmic lactate retention in Colo357 spheroids (diameter ~150 μm). MiaPaCa2 cells, which are Cx43 negative in monolayer culture, showed markedly increased Cx43 immunoreactivity at areas of invasion in orthotopic xenograft mouse models. These tissue areas were associated with chronic extracellular acidosis (as indicated by the marker LAMP2 near/at the plasmalemma), which can explain the advantage of junctional transmission over MCT *in vivo*. We propose that Cx43 channels are important conduits for dissipating lactate anions from glycolytic PDAC cells. Furthermore, lactate entry into the better-perfused recipient cells has a favourable alkalinizing effect and supplies substrate for oxidative phosphorylation. Cx43 is thus a novel target for influencing metabolite handling in junctionally-coupled tumours.

## Introduction

Tumours produce large amounts of lactate,^1^ which, in the absence of oxidative phosphorylation, is a waste product that must be removed. The best characterized venting mechanism involves monocarboxylate transporters (MCT) that translocate lactate and H^+^ ions across the cell membrane.^2, 3, 4, 5^ However, solid tumours commonly develop a profoundly acidic extracellular microenvironment,^6, 7, 8, 9, 10, 11^ which thermodynamically hinders H^+^-coupled lactate efflux. It is therefore unclear how MCTs could produce an adequate export of lactate anions under these circumstances. Alternative routes for discharging lactate may be necessary to support glycolytic cancer metabolism. Without a complete characterization of all lactate-handling pathways, full efficacy of therapies aimed at attenuating lactate removal^12^ cannot be achieved.

An alternative route for venting lactate anions from poorly perfused cells may involve gap junctions assembled from connexin proteins.^13^ These channels couple the cytoplasm of neighbouring cells into a syncytium and establish high-conductance pathways for the passage of small molecules. Since direct cell-to-cell junctional flux is thermodynamically insensitive to extracellular pH (pH_e_),^14^ it may compensate for reduced MCT activity in acidic microenvironments. As yet, this junctional route has not been considered as a means of handling cancer metabolites because of the widely held notion that gap junctions are not associated with cancer,^15^ and act as tumour suppressors in at least some types of neoplastic disease.^16, 17, 18^ However, several reports have produced evidence that connexins may, in fact, facilitate late-stage disease in certain cancers.^19, 20, 21, 22, 23^

Pancreatic ductal adenocarcinoma (PDAC) tumours, a highly invasive cancer, are characterized by profoundly under-perfused regions^24, 25^ which require efficient mechanisms for off-loading lactate into the nearest functional blood vessel. Genes coding for connexins are expressed in many PDAC cell lines,^26^ and we hypothesize that gap junctions are important conduits for transmitting lactate across clusters of PDAC cells, towards regions that have the most favourable transmembrane gradient for MCT-assisted off-loading. We produce evidence that junctional transport of lactate benefits tumours dually by (i) offering a pH_e_-insensitive route for discharging metabolic waste from glycolytic cells, and (ii) alkalizing recipient cells to levels that are generally conducive for proliferation.^27, 28, 29^ Our findings propose a novel, lactate-handling role for Cx43 which may be particularly critical in acidotic regions of tumours.

## Results

### PDAC cell lines differ in their gap junctional connectivity

The canonical pathway for removing lactate involves MCT proteins. Expression of MCT isoforms 1 and 4 was measured by immunoblotting in cell lines^30, 31^ established from primary PDAC tumours (MiaPaCa2, BxPC3, PANC1), ascites (AsPC1) and metastatic lesions (Colo357, Capan1) (Figure 1a). MCT1 was detected in all cell lines, and hypoxia-inducible MCT4 was strongly expressed in all except Capan1. A possible alternative to MCT-dependent venting is cell-to-cell lactate transmission via connexin channels. A published microarray dataset of 22 human PDAC cell lines established from either primary tumour or metastatic lesions^26^ demonstrates expression of genes coding for connexins (Figure 1bi). Among these, the most prominently expressed genes code for Cx43, Cx26 and Cx45 (genes: *GJA1*, *GJB2* and *GJC1*). Colo357, BxPC3 and MiaPaCa2 were selected for further studies on the basis that these represent various combinations of *GJA1*, *GJB2* and *GJC1* expression (Figure 1bii).

At protein level, Cx43 was present in Colo357 and BxPC3 cells, but not in MiaPaCa2 cells *in vitro*. Cx45 was present in MiaPaCa2 only, whereas Cx26 was negligible in all three lines (Figure 1ci). Activation of hypoxic signalling (by incubation with DMOG or 2% O_2_) raised Cx43 levels in BxPC3 cells (Figure 1c). To determine how connexin immunoreactivity relates to cell-to-cell coupling, junctional permeability to the fluorescent marker calcein (*P*_junc,calc_) was measured from fluorescence recovery after photobleaching (FRAP; Figure 1cii). Calcein-loaded confluent monolayers were imaged and fluorescence in a central cell was bleached to 50% of initial intensity. Recovery of fluorescence due to calcein diffusion from neighbouring cells quantified *P*_junc,calc_; this was highest in Colo357 monolayers, moderate in BxPC3 monolayers yet raised by hypoxic signalling, and very low in MiaPaca2 monolayers (Figure 1cii). Thus, cell-to-cell diffusive coupling correlated with Cx43 levels. Transmission was blocked by the gap junctional inhibitor carbenoxolone (CBX; 100 μm). To test that Cx43 is responsible for assembling the conduits for calcein, *GJA1* knockdown was performed in Colo357 cells transduced with shRNA constructs. Compared with the scrambled control, the shRNA construct with the greatest knockdown efficacy (construct #1) reduced Cx43 immunoreactivity by 70% (Figure 1di) and reduced *P*_junc,calc_ by >90% (Figure 1dii), confirming Cx43 to be a major gap junction-forming protein. Construct #2 had lower knockdown efficiency (45%) and produced a smaller decrease in *P*_junc,calc_ (70%). *GJA1* knockdown did not change the expression of MCT1 or MCT4 (Supplementary Figure S1), indicating that MCT-dependent lactate handling is unaffected by genetic ablation of junctional coupling.

### Lactate anions permeate gap junctions faster than heavily buffered H^+^ ions

Previous studies^32^ have measured cytoplasmic lactate diffusivity (*D*_lac_) in tumours to be ~1000 μm^2^/s. In contrast, lactate permeability through gap junctions connecting cancer cells (*P*_junc,lac_) is poorly characterized. Direct *P*_junc,lac_ measurements would require a means of evoking a local rise of intracellular [lactate] ([lactate]_i_), but such manoeuvres are not easily implemented. However, as small (≪1 kDa) ions permeate gap junctions by a diffusive process, a constant of proportionality can be used to convert diffusivity measurement into permeability constants (for example, *D*_lac_ into *P*_junc,lac_). Conveniently, this proportionality constant can be determined by studying the dynamics of H^+^-binding small molecules, commonly referred to as mobile buffers. Photolytic H^+^-uncaging from the membrane-permeable donor 6-nitroveratraldehyde (NVA) drives the diffusive exchange of protonated for unprotonated mobile buffers,^33^ which can be imaged using pH-sensitive cSNARF1 fluorescence to derive cytoplasmic H^+^ diffusivity (*D*_H_) and junctional H^+^ permeability (*P*_junc,H_) (Figure 2a). Among the cytoplasmic H^+^-buffers, CO_2_/HCO_3_^−^ is unusual because CO_2_ permeation across membranes can short-circuit the junctional route. Thus, to characterize only those H^+^-buffers that exchange between cells via gap junctions, experiments were performed using CO_2_/HCO_3_^−^-free (Hepes-buffered) superfusates. *D*_H_ measurements were made on MiaPaCa2, BxPC3 and Colo357 cells (Figure 2bi). H^+^-uncaging at one end of a cell produces a local cytoplasmic build-up of H^+^ ions which then spreads diffusively. The cytoplasm of all three PDAC lines had comparable *D*_H_ (Figure 2bii). To measure *P*_junc,H_, H^+^ ions were released in a larger region of a cell at the middle of a confluent monolayer, and their spread into neighbouring cells was imaged (Figure 2ci). *P*_junc,H_ was highest in Colo357 cells, moderate in BxPC3 cells (but increased by hypoxic signalling) and very low in MiaPaCa2 cells (Figure 2cii). CBX (100 μM) inhibited transmission in coupled wild-type cells, as did *GJA1* knockdown with shRNA #1, relative to Colo357 cells transduced with scrambled construct (Figure 2cii). Thus, by using the experimentally determined *P*_junc,H_/*D*_H_ ratio to approximate *P*_junc,lac_/*D*_lac_, *P*_junc,lac_ was estimated to be ~1 μm/s in Colo357 and hypoxic BxPC3 monolayers.

To confirm that lactate anions penetrate gap junctions faster than highly-buffered H^+^ ions, Colo357 monolayers were exposed to a standing gradient of extracellular [lactate] ([lactate]_e_) maintained by a double-barrelled micropipette^34^ (Figure 2di). MCT-dependent H^+^-lactate entry into cells under the lactate-containing microstream is reported as an intracellular acidification; this produces a smooth longitudinal gradient of pH_i_ between cells exposed to the lactate-containing microstream and cells under the lactate-free microstream (Figure 2di). At steady-state, the size of this longitudinal gradient of pH_i_ depends on junctional fluxes of H^+^ and lactate ions (Figure 2dii). If junctional transmission of lactate anions were faster than the highly-buffered H^+^ ions, rapid syncytial dissipation of lactate would allow further lactate (and H^+^) entry into cells under the lactate-containing microstream. As net MCT activity continues until equilibriation ([H^+^]_i_ × [lactate]_i_ = [H^+^]_e_ × [lactate]_e_), a more dissipated syncytial [lactate]_i_ gradient is predicted to evoke a steeper pH_i_ gradient. Indeed, the experimentally measured pH_i_ gradients were larger when gap junctions were open, compared to when they were blocked with CBX (100 μm; Figure 2diii) or ablated genetically by knockdown (shRNA #1; Figure 2div). Thus, unlike MCT which transports H^+^ and lactate ions in strict 1:1 stoichiometry, junctional lactate permeability exceeds junctional H^+^ permeability, at least in Colo357 monolayers under CO_2_/HCO_3_^−^-free conditions.

### Gap junctions can transmit a substantial traffic of lactate in PDAC spheroids

A glycolytic cell may discharge lactate via gap junctions or by MCTs, depending on the magnitude of junctional lactate permeability (*P*_junc,lac_) and lactate permeability via MCT (*P*_mct,lac_). *P*_mct,lac_ was measured in cSNARF1-loaded Colo357 monolayers exposed rapidly to lactate-containing solution. Uniform superfusion collapses any cell-to-cell gradients and thus eliminates net junctional flux. The experiment shown in Figure 3a, performed in CO_2_/HCO_3_^−^-containing buffer, derives the magnitude of MCT flux (J_mct_) from the product of the rate of pH_i_ change and buffering capacity (intrinsic plus CO_2_/HCO_3_^−^-dependent buffering; Supplementary Figure S2). As intracellular carbonic anhydrase activity in Colo357 cells is low (Supplementary Figure S3), CO_2_/HCO_3_^−^ reaction kinetics were implemented in the calculation.^35^ The relationship between *J*_mct_ and *P*_mct,lac_ is given as *J*_mct_= *P*_mct,lac_ × *ρ* × [lactate]_e_, where *ρ* is the surface area/volume ratio. For a Colo357 monolayer, *ρ* is the reciprocal of monolayer height which was estimated from the cells’ area in the *x*–*y* plane (592±34 μm^2^) and volume measured separately by flow cytometry (3570±69 μm^3^). Thus, P_mct,lac_ in Colo357 cells was 0.3 μm/s (Figure 3b), which is smaller than *P*_junc,lac_ (~1 μm/s). For note, the junctional blocker CBX had a small, off-target inhibitory effect on *P*_mct,lac_.

A direct comparison between junctional and MCT-mediated lactate transport can be made in spheroid tissue growths, where radial [lactate]_i_ gradients are able to drive junctional fluxes. Although lactate sensors^36^ can report [lactate]_i_ dynamics, they are unable to distinguish whether lactate is transported by MCT or through gap junctions. The route taken by lactate can, however, be inferred from its pH_i_-signature. Lactate entering a cell by MCT carries with it an H^+^ ion, producing acidification; in contrast, junctionally transmitted lactate diffuses faster than heavily buffered H^+^ ions, evoking the opposite (that is, alkalinizing) pH_i_-response. Colo357 spheroids grown to a radius of ~110 μm develop modest core-hypoxia, and can be imaged confocally for pH_i_ across the equatorial plane (Figure 3ci). At the spheroid periphery, the ‘lactate addition/removal’ protocol evoked a pH_i_ response that was similar to that recorded in monolayers (Figure 3cii). The direction of this pH_i_ change indicates that most lactate traffic in peripheral cells is carried by MCT. With depth, diffusion across the tortuous extracellular space becomes increasingly rate-limiting for MCT-mediated transport (tortuosity was estimated to reduce diffusivity by 70% Supplementary Figure S4). Reduced MCT fluxes may unmask junctional transmission occurring across the larger, syncytial cytoplasmic volume. Indeed, the pH_i_ response to lactate addition/removal measured at the spheroid core was biphasic (Figure 3cii; further analysis in Figure 3d). This direction of the initial pH_i_ transients is thermodynamically inconsistent with MCT activity; instead, it indicates that a significant fraction of lactate traffic to and from the spheroid core is routed through gap junctions (Figure 3aiii). In support of the involvement of Cx43 channels, blocking gap junctions with CBX eliminated the initial pH_i_ transients (Figure 3d).

To further investigate the two routes for lactate handling at the core of spheroids, the balance between MCT and junctional fluxes was manipulated experimentally. In the absence of CO_2_/HCO_3_- buffer, MCT flux can be modulated by changing the concentration of Hepes, a membrane-impermeant buffer which carries H^+^ ions towards and away from MCT proteins.^37^ MCT activity in cells that are furthest away from the bulk superfusate will be most sensitive to a reduction in [Hepes], whereas cells at the spheroid rim (with good diffusive coupling) are the least sensitive. Superfusion with 20 mm Hepes provides a high level of mobile buffering that greatly facilitates extracellular H^+^ diffusivity. Under this buffering regime, lactate addition evokes prompt intracellular acidification at the periphery and at the core, indicative of a major component of MCT-dependent entry throughout the spheroid. Reducing [Hepes] to 1 mm substantially weakens diffusive coupling, particularly with the spheroid core. In these low-buffer conditions, lactate addition evoked an intracellular alkalinization at the spheroid core (Figure 3ei) and lactate removal acidified the core (Figure 3eii), both of which are consistent with junctional transmission. Furthermore, these transient pH_i_ responses were ablated in *GJA1* knockdown spheroids (shRNA #1; Figure 3f). At the spheroid rim, where diffusion distances are short, pH_i_ responses were less sensitive to a reduction in [Hepes] (that is, MCT activity remained fast).

Junctional and MCT-mediated lactate fluxes (*J*_junc_, *J*_mct_) at the core of spheroids can be quantified from the rates of pH_i_ change (dpH_i_/d*t*). In spheroids treated with CBX (to block *J*_junc_), dpH_i_/d*t*_CBX_ is proportional to −*J*_mct_ (corrected for the partial inhibitory effect of CBX on MCT; Figure 3b). In junctionally coupled spheroids, dpH/d*t*_con_ reports *J*_junc_−*J*_mct_. Thus, the fraction of lactate traffic that is routed through gap junctions is:


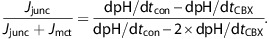


This equation estimates that 77±3% of lactate flux at the core of in Colo357 spheroids was carried junctionally. At the core of spheroids grown from Cx43-positive NHDF-Ad fibroblasts and HEK293.T kidney cells, the fraction of lactate carried junctionally was 50±5% and 30±4%, respectively (Supplementary Figure S5). Thus, gap junctions are an important route for transporting lactate in Cx43-expressing tissue growths.

### Junctional discharge of lactate from hypoxic cells alkalinizes the normoxic rim of PDAC spheroids

Resting pH_i_ is set by the balance of all acid-base fluxes and is 7.126±0.0075 (s.e.m.; *N*=250 cells) in Colo357 monolayers. In 3D tissue growths, junctional flux of lactate (a chemical base) may exert an additional influence on the resting pH_i_ of recipient cells. This was studied in Colo357 spheroids (Figure 4a). In spheroids grown in glucose-containing media, resting pH_i_ in the outermost layer was ~0.3 pH units more alkaline than in monolayers (Figure 4b). Replacing glucose with galactose (to block lactate production^38^) reduced the degree of peripheral alkalinization (Figure 4b). Thus, the highly alkaline spheroid rim is related to metabolic lactate production. Spheroids grown from Cx43-knockdown cells (construct #1; Figure 1d) were less alkaline at the rim compared to control spheroids (scrambled construct), indicating that the junctional route carries the aforementioned alkalinizing flux (Figure 4c). In support of this, treatment of wild-type spheroids with CBX (100 μm; 3 h) blocked peripheral alkalinization (Figure 4d). CBX also acidified the spheroid core, which is likely due to the partial inhibitory effects of CBX on MCT activity (Figure 3b). In summary, the junctional spread of intracellular lactate from the spheroid core alkalinizes the outermost cells (Figure 4e). This effect is able to take place because the junctional flow of lactate is uncoupled from the flow of H^+^ ions that are also produced by glycolytic metabolism (Figure 4e).

### Junctional coupling reduces intracellular lactate retention in PDAC spheroids

Junctional transmission of metabolites would benefit under-perfused tissues by giving all cells access to regions with the most favourable transmembrane gradient for off-loading lactate by MCTs, which would normally be at the normoxic rim. Inactivation of junctional lactate flux is therefore expected to increase lactate retention in the intracellular compartment. This was tested by comparing steady-state lactate levels in intracellular fluids and in samples of medium. Colo357 cells were grown as monolayers (that is, minimal diffusion distances) or spheroids (structures with restricted diffusion). After a set period of growth, aliquots of media and cell lysates were prepared for measurements of [lactate], as well as of [glucose], osmolarity and protein that are required for calculating intracellular [lactate] ([lactate]_i_), as explained in the Methods (Figure 5a). To test if transduction with shRNA constructs had affected lactate production, wild-type and genetically modified Colo357 monolayers were grown in glucose-containing media. After 4 days of growth, the [lactate]/protein ratio in lysates of wild-type Colo357 cells and cells transduced with scrambled shRNA, shRNA #1 and #2 were comparable (Figure 5b). This result indicates that the genetic ablation of *GJA1* does not affect glycolytic rate in 2D culture. As confirmation that the source of lactate is glycolytic, wild-type cells incubated with galactose-containing media produced no detectable [lactate] (Figure 5b). The longer extracellular diffusion distances inside spheroids are expected to increase intracellular lactate retention, reported as the [lactate]_i_/[lactate]_e_ ratio. Wild-type spheroids as large as ~75 μm in radius were able to vent lactate as efficiently as monolayers (Figure 5c). However, intracellular lactate retention increased in larger spheroids (Figure 5c). To test if the ability of ~75 μm spheroids to minimize lactate retention is related to junctional coupling, measurements were performed on *GJA1* knockdown spheroids (shRNA #1). Compared to scrambled controls (matched for growth period), lactate retention was substantially greater in knockdown spheroids (Figure 5d), an observation that cannot be explained by metabolic rate (Figure 5b) or MCT expression (Supplementary Figure S1). Lactate retention increased in larger spheroids, but junctionally coupled tissues were able to grow at a faster rate, which may relate to better lactate venting. The proposed mechanism for Cx43-facilitated lactate venting from tissues is illustrated in Figure 5e.

### *GJA1*-knockdown Colo357 cells are less successful in colonizing the proliferating rim of spheroids

Gap junctions provide a route for dissipating lactate which benefits a syncitium of glycolytic cells (Figure 5e). In addition, junctional transmission of lactate alkalinizes recipient cells, producing a milieu chemistry that is conducive for growth (Figure 4e). Thus, cells with high Cx43 coupling are predicted to be more successful in areas of proliferation. Competition for space between Colo357 cells expressing various levels of Cx43 activity was tested in spheroid co-culture models; the more successful phenotype is expected to expand in the proliferating rim of spheroids. Spheroids were grown from a 1:1 mixture of Cx43-positive wild-type Colo357 cells and *GJA1*-knockdown Colo357 cells (shRNA #1 and #2, Figure 1d). The knockdown cells were identified by eGFP fluorescence. The upper panels in Figure 6a show transmission images superimposed with eGFP fluorescence measured across the equatorial plane of the co-culture spheroid. The lower panels analyse fluorescence relative to the mean signal across the imaging plane (above-average pixels shown as red and below-average pixels appearing blue). Wild-type/shRNA #1 spheroids showed a clear segregation of cells by Cx43 levels (Figure 6a): wild-type cells (Cx43-positive) were abundant in the proliferating rim, typically along a crescent; in contrast, *GJA1*-knockdown cells clustered at the core. A similar observation was made with wild-type/shRNA #2 spheroids (Figure 6b). As a control, co-culture of wild-type cells with cells expressing the scrambled construct produced a random pattern of fluorescent and non-fluorescent patches, indicating no clear advantage of either phenotype in populating the proliferating rim (Figure 6c). Fluorescence images were quantified further in Figure 6d as a function of distance from the spheroid surface. In summary, higher Cx43 activity is an advantage for Colo357 cells competing for space in the proliferating rim of spheroids.

### Cx43 immunoreactivity is increased in invading PDAC cells *in vivo*

A number of PDAC lines, including MiaPaCa2, do not express Cx43 *in vitro* when grown as monolayers (Figures 1bii and ci). Under uniform culture conditions, monolayers are unable to benefit from junctional lactate flux, which may explain the redundancy of Cx43 function. Nonetheless, a lactate-venting role for gap junctions may become apparent *in vivo*. To test this, orthotopic xenografts were established from MiaPaCa2 cells in the proximal part of the pancreas of nude mice. Sections of primary tumour excised together with normal pancreatic tissue, duodenum and stomach, as well as sections of lung were stained for human Cx43 or human epidermal growth factor receptor (EGFR) as a human PDAC marker. To identify co-localization between Cx43 and EGFR immunoreactivity, image pairs were registered by matching histological landmarks, filtered by colour to identify positive staining and then overlaid. The EGFR-positive primary tumour showed no evidence for Cx43 immunoreactivity (Figure 7a; showing as blue on overlay), which phenocopies monolayer culture *in vitro*. In most cases of lung metastases, Cx43 staining was weak or absent (Figure 7b), but some examples of metastatic lesions were Cx43 positive (Figure 7c). Markedly increased Cx43 immunoreactivity was observed in MiaPaCa2 cells invading host pancreatic tissue (Figure 7d), and also in clusters of MiaPaCa2 cells invading the duodenal mucosa (Figure 7e) and the stomach muscularis externa (Figure 7f; co-localization of Cx43 and EGFR staining indicated by purple pixels on overlay). Cx43 staining was concentrated at the surface membrane, where the protein is able to assemble into cell-to-cell conduits (Supplementary Figure S6). In 13 sections obtained from four mice, out of 547 clusters of EGFR-positive MiaPaCa2 cells, 109 clusters (20%) were strongly positive for Cx43 (Supplementary Figure S7A). The appearance of Cx43 staining in these clusters was predominantly uniform, arguing that these may have been established from a Cx43-positive clone. These findings may suggest that Cx43-positive cells are more invasive or better adapted for colonizing new niches. Increased Cx43 staining at the tumour/pancreas interface was also observed with orthotopically implanted PANC1 cells, another PDAC cell line with modest *in vitro* Cx43 expression (Supplementary Figure S8).

Chronic extracellular acidosis in under-perfused areas occupied by PDAC cells may be a factor favouring the junctional route over the pH_e_-sensitive MCT mechanism. A recent study^9^ has shown that over-expression and redistribution of lysosome-associated membrane protein 2 (LAMP2) towards the plasma membrane is a marker of acidotic niches. Results from four mice demonstrate that LAMP2 staining was diffuse in primary tumour regions (Figure 8ai and aii), as expected for the protein’s normal lysosomal targeting. In contrast, invasions of the duodenum mucosa (Figure 8bi) and stomach muscularis (Figure 8bii) contained a subpopulation of MiaPaCa2 cells in which LAMP2 staining was concentrated near or at the plasma membrane. Analysis of 17 sections obtained from four mice showed LAMP2 staining at or near the plasmalemma in 39% of clusters containing MiaPaCa2 cells (Supplementary Figure S7B). In these niches, the acidic extracellular milieu would favour junctional lactate transmission.

## Discussion

Enhanced anaerobic glucose metabolism favours PDAC progression,^24^ but sustaining an elevated glycolytic rate requires adequate pipelines for discharging lactate. PDAC cells express MCT1 and MCT4 proteins (Figure 1a),^24^ which can shuttle lactate out of cells, provided that the transmembrane [lactate] and pH gradients are favourable. Through a process called metabolic symbiosis,^2, 39^ stromal cells can absorb extracellular lactate and hence facilitate lactate off-loading from glycolytic cancer cells. However, the extracellular milieu of solid tumours is commonly more acidic than cytoplasm^6, 7, 8, 9, 10, 11^ and this curtails MCT-dependent lactate efflux from cancer cells. *In vivo* evidence for this pH-related thermodynamic constraint is suggested by the appearance of the acidosis marker LAMP2^9^ near or at the plasma membrane of invading PDAC cells (Figure 8). Strong diffusive coupling between PDAC cells attained by means of Cx43 channels (for example, Colo357, hypoxic BxPC3 or invading MiaPaCa2) provides an alternative route for exchanging metabolites across cellular networks. The magnitude of lactate traffic discharged junctionally and aboard MCTs can be compared in PDAC spheroids by exploiting the observation that these two fluxes evoke opposite pH_i_ responses (Figure 3). This difference is because MCT activity enforces a 1:1 stoichiometry between lactate and H^+^ ions, whereas lactate anions penetrate junctions faster than highly-buffered H^+^ ions (Figure 2d). In Colo357 spheroids, as many as 4-in-5 lactate anions are vented junctionally from the core (Figure 3), and a sizeable traffic of lactate through gap junctions was also recorded in non-PDAC spheroids (Supplementary Figure S5). Junctional transmission of lactate does not produce net electric current because connexin channels are also highly permeable to monovalents (for example, K^+^, Cl^−^ present at ~100 mm), which promptly neutralize any potential difference between cells. Coupling by connexins improves overall lactate venting from clusters of cells by allowing lactate anions to dissipate across the cytoplasmic syncytium towards regions where off-loading by MCT is thermodynamically most favourable (that is, nearest to a blood vessel). This was confirmed by biochemical assays, which demonstrated a smaller degree of intracellular lactate retention in junctionally coupled tissue growths (Figure 5d).

The dissipation of lactate anions (a chemical base) across the syncytial cytoplasm, down a gradient maintained by oxygen tension, has an alkalinizing effect on recipient cells (Figure 4). As this shift in pH_i_ comes at no energetic cost to recipient cells, it is favourable over secondary active pH_i_ regulators. Since high pH_i_ is permissive for growth,^27, 28, 29^ proliferation in peri-hypoxic tumour regions could be stimulated by coupling onto lactate-supplying cells. An additional benefit of junctional lactate transmission is that it supplies normoxic cells with substrate for oxidative phosphorylation without having to rely on MCT activity. This is significant because metabolic symbiosis between lactate-generating hypoxic cells and lactate-catabolizing normoxic cells^2^ facilitates PDAC progression.^24^

The role of gap junctions in transporting metabolites between cells is likely to have been understated in the past as a consequence of experimental designs based largely on monolayer culture. In monolayers exposed uniformly to culture media or superfusates, cell-to-cell solute gradients are collapsed and net junctional transmission cannot be observed. However, uniformity of solute concentration is not representative of tumours, where heterogeneity of pH and oxygenation establishes gradients that can drive metabolites through gap junctions.

Gap junctions are assembled from the protein products of at least 20 connexin genes, but the most commonly expressed isoforms in PDAC cells are Cx43, Cx45 and Cx26 (Figure 1bi). High Cx43 expression in cells such as metastatic Colo357 produced strong cell-to-cell coupling (Figure 1c). Comparable coupling and Cx43 expression were attained under hypoxia in BxPC3 cells (Figure 1). This hypoxic response may involve the binding of hypoxia-inducible factor to a putative hypoxia response element (GGCGTGAGG)^40^ at 5094–5098 base pairs downstream of the start codon of *GJA1*. In contrast, hypoxic induction of Cx43 was not observed *in vitro* in MiaPaCa2 cells. Increased Cx43 positivity was, however, described in a subpopulation of MiaPaCa2 cells spreading into the host pancreas and at invasions of the duodenum and stomach of orthotopic PDAC mouse models, where one-in-five PDAC clusters were Cx43 positive (Figure 7). Chronic extracellular acidosis, demonstrated by the appearance of LAMP2 at or near the plasmalemma in 39% of invading clusters (Figure 8b), may explain the selective advantage of Cx43 channels over MCTs. On the basis of our junctional lactate handling model, we propose that Cx43-positive cells are better at colonizing new niches. This hypothesis is supported by the finding that Colo357 cells with high Cx43 levels segregated preferentially at the proliferating rim of spheroids (Figure 6). The metabolic advantage may explain the significant upregulation of Cx43 reported by others in three pancreatic cancer datasets: Bandea (3.5-fold, *P*=10^−9^), Segara (4.1-fold, *P*=0.001) and Buchholz (1.6-fold, *P*=0.02; see Supplementary Information). Furthermore, higher Cx43 expression (3.6-fold, *P*=0.003) has been associated with tumours sensitive to inhibitors of EGFR.^41, 42, 43^

In summary, our results propose a novel metabolic role for Cx43-assembled gap junctions in handling lactate in networks of cancer cells. This junctional venting mechanism can be important in lieu of the canonical MCT-operated mechanism that is inhibited by the acidic tumour microenvironment. Considering the necessity of supporting a high glycolytic rate, Cx43 channels may be particularly advantageous for highly-proliferating cells at invasion fronts and distant metastases. Therapeutic approaches targeting lactate handling in tumours^44^ need to be re-evaluated in light of the proposed junctional route in connexin-positive cancers.

## Materials and methods

### Connexin expression data

Microarray data for connexin isoform gene expression in 22 PDAC cell lines was obtained from http://biogps.org (dataset E-GEOD-21654). Gene expression in pancreatic tumours was obtained from the Pancreatic Expression Database (http://www.pancreasexpression.org/).

### Cell lines

AsPC1, BxPC3, Capan1, MiaPaCa2, PANC1 and Colo357 were kindly provided by Holger Kalthoff (Kiel, Germany). NHDF-Ad fibroblasts were purchased from Lonza. HEK293.T cells were a kind gift from Adrian Harris (Oxford, UK). Cells were authenticated by STR profiling and tested for mycoplasma. AsPC1, BxPC3, MiaPaCa2, PANC1, Colo357, NHDF-Ad and HEK293.T cells were grown in bicarbonate-buffered DMEM (Sigma-Aldrich, Dorset, UK; D6546) containing 10% FBS (Sigma-Aldrich, F9665; Capan1 cells in 20%), 2 mm l-glutamine, 20 U/ml penicillin and 20 μg/ml streptomycin (Sigma-Aldrich) and 0.1 mg/ml Normocin (InvivoGen, San Diego, CA, USA). Cells were trypsinised with 0.1% Trypsin (Gibco, Bleiswijk, Netherlands, 15400-054) and passaged twice per week. Monolayers were plated in IBIDI or LabTek chambers (seeded at 50 000 and 70 000 cells, respectively) for real-time imaging, in 6 cm dishes (seeded at 600 000 cells) for 4 days for collecting adequate cell mass for biochemical assays, or in 6 cm dishes (seeded a 1 200 000 cells) for 3 days for immunoblotting. For pH imaging and co-culture experiments, spheroids were cultured using the hanging-drop method (400 Colo357 cells per 20 μl, 400 HEK293.T cells per 20 μl, 2000 NHDF-Ad cells per 20 μl) for 3 days. For biochemical assays, spheroids were grown in spinner flasks in 250 ml medium, seeded at 5 000 000 cells, for up to 120 h. MiaPaCa2 and PANC1 cells used for xenografting were cultured in DMEM/F12 with Glutamax, 10% FCS and 2.5% horse serum, and in high-glucose DMEM with 10% FCS, respectively. 25 mm galactose-containing medium was based on glucose-free DMEM; all other media contained 25 mm glucose.

### *GJA1* knockdown

HEK293T packaging cells were plated into 25T T/C flasks and grown to 50% confluency, and transfected (CaPO_4_ method) with packaging plasmids pSPAX2, pMD2.G and shRNA construct (Genecopoiea, HSH007411-1-LVRU6GP, HSH007411-2-LVRU6GP, HSH007411-3-LVRU6GP and HSH007411-4-LVRU6GP; CSHCTR001-LVRU6GP was the scrambled control). Lentivirus-containing media were collected, filtered through 0.45 μm syringe filter and added to PDAC cells for 72 h. Transduced cells were selected with puromycin (6 μg/ml).

### Immunoblotting

To measure protein levels under normoxic conditions, cells were incubated in an atmosphere of 21% O_2_. To characterize the effect of stabilizing hypoxic signalling, cells were incubated with dimethyloxalylglycine (1 mm DMOG; in 0.1% DMSO) or incubated in 2% O_2_ for 48 hours. Cells were then lysed in RIPA buffer containing protease/phosphatase inhibitors. Overall, 55 μg of total protein (BCA assay, Thermo, Rockford, IL, USA) was loaded per lane, separated using polyacrylamide gel electrophoresis and transferred onto PVDF membrane. Membranes were blocked in 5% skimmed milk and incubated with primary antibodies raised against human MCT1 (Millipore, Darmstadt, Germany, AB 3538P), MCT4 (Novus, Littleton, CO, USA, NBP1-00920), Cx43 and β-actin (Cell Signaling Technologies, Danvers, MA, USA, 3512, 5125), Cx26 and Cx45 (Abcam, Cambridge, UK, AB65969, AB78408) and mouse monoclonal antibody against human CAIX (M75, kind gift from Silvia Pastorekova, Bratislava, Slovakia). Membranes were then incubated with HRP-conjungated secondary antibodies (Novus NB7256, NB7185 or NB120-7061) and developed using ECL (Thermo). Blots were repeated three times on independent lysates.

### Solutions and superfusion

5% CO_2_/HCO_3_^−^-buffered solution (in mm): 125 NaCl, 4.5 KCl, 11 glucose, 22 NaHCO_3_, 1 CaCl_2_, 1 MgCl_2_. Hepes-buffered solution: NaHCO_3_ replaced with 20 mm or 1 mm HEPES, and NaCl raised to osmolarity of 300 mOsm/kg. Lactate-containing solution: NaCl replaced with NaLactate. Ammonium-containing solution: NaCl replaced with NH_4_Cl. pH adjusted to 7.4 with 4 m NaOH. Monolayers were superfused in the LabTek or IBIDI chambers. Spheroids were superfused in a Perspex chamber with a poly-l-lysine treated coverslip. Dual microsuperfusion of monolayers using a double barrel micropipette is described previously.^34^

### Confocal imaging

Cells were loaded with 10 μg/ml cSNARF1-AM (5 min for monolayers and up to 3 h for spheroids) or 4 μm calcein-AM (5 min). cSNARF1 fluorescence was excited at 555 nm, measured at 580 nm/640 nm and converted to pH using a calibration curve determined using the nigericin method. Calcein fluorescence was excited at 488 nm laser and measured >510 nm. Imaging was performed on a Zeiss LSM system (Jena, Germany). Pinhole for monolayer and spheroid experiments was 2.9 and 1.8 Airy units, respectively. Measurements on individual cells or cell clusters were repeated on different monolayers; measurements on spheroids were repeated at least four times on different batches seeded independently.

### Mathematical algorithms

(a) FRAP analysis: Calcein fluorescence was normalized to signal in remote regions of the monolayer. Signal in the central (bleached) cell was best-fitted to a mono-exponential function to estimate the time constant *τ*. Calcein permeability, *P*_junc,calc_ (units: μm/s), was calculated as the perimeter of the central cell × area of central cell/*τ*. (b) Cytoplasmic H^+^ diffusivity (*D*_H_): [H^+^] was measured in ten regions of interest, offset to baseline, and fitted to a constant-source diffusion equation for over 30 s:


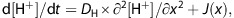


where *J* is the rate of H^+^ uncaging. (c) Junctional H^+^ permeability, *P*_junc,H_. Mean [H^+^] was measured in the central cell and in all directly neighbouring cells. *P*_junc,H_ was calculated by best-fitting, over 60 s, to a system of equations for [H^+^] in the central ([H^+^]_c_) and neighbouring ([H^+^]_n_) cells:


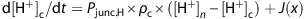



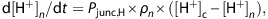


where *ρ*_c_ is the perimeter/area ratio of the central cell and *ρ_n_* is the ratio of the central cell perimeter to the area of the ensemble of neighbouring cells.

### Orthotopic xenografts

Animal experiments were approved by the administration of Lower Saxony (Germany). Animals were handled according to national guidelines (license 33.9-42502-04-13/1085). Orthotopic PDAC xenografts were obtained by transplantation of human MiaPaCa2 or PANC1 cells (10^6^ cells in 20 μl PBS) into the proximal part of the pancreas of anaesthetized (15 mg/kg xylazine, 75 mg/kg ketamine) 10–20 week NMRI Fox^nu/nu^ nude male mice (Charles River, Sulzfeld, Germany) as described previously.^45^ Mice were sacrificed after ~90 days (MiaPaCa2) or ~115 days (PANC1) and only those that developed xenografts were used for sectioning. Lungs and pancreatic tumour excised with the stomach, duodenum and the rest of the pancreas were fixed in 4% formalin in PBS overnight before embedding in paraffin. For each cell line tested, multiple tissue sections were obtained from four animals in order to accurately establish the fraction of clusters that were positive for a tested epitope among a large number of PDAC-positive clusters. No comparisons were made between xenografts of different cell lines. Animal randomization was not required.

### Immunohistochemical staining

Deparaffinised and rehydrated 2-μm sections of tissues were pre-treated at 98 °C for 20 min in citrate buffer (pH 6, Dako, Glostrup, Denmark) and incubated with 3% H_2_O_2_ for 10 min. For Cx43 and EGFR staining, sections were blocked with SEA BLOCK buffer (Thermo) for 20 min and directly incubated with the polyclonal rabbit anti-Cx43 antibody (Cell Signaling Technologies, 1:100) or with the monoclonal rabbit anti-human EGFR antibody (Life Technologies, 1:100; MA5-16359) overnight at 4 °C. For LAMP2 staining, tumour sections were incubated for 60 min with Mousestain kit reagent A (v-histofine, Nichirei Biosciences, Tokyo, Japan) and directly incubated with the monoclonal mouse anti-LAMP2 antibody (Abcam; ab119124, cl. H4B4, 1:200). Anti-Cx43 and anti-EGFR primary antibodies were revealed by Simple Stain rabbit Max PO (v-histofine, Nichirei Biosciences). Anti-LAMP2 antibody was revealed by reagent B and Simple Stain mouse Max PO of Mousestain Kit (Nichirei Biosciences). AEC staining was performed for 30 min and then sections were washed in water and counterstained for 20 s with Meyers hematoxylin. Sections were washed twice for 5 min in TRIS buffer between steps. To generate overlays, image pairs stained for EGFR and Cx43 were aligned (‘registered’) using 10–20 histological landmarks (ImageJ plugin: http://imagej.net/Landmark_Correspondences rigid non-linear least squares method). Staining patterns were extracted by filtering colour (IHC toolbox, https://imagej.nih.gov/ij/plugins/ihc-toolbox/index.html) and pseudocoloured to distinguish the Cx43 and EGFR channels (red and blue, respectively). Image pairs were overlaid for presentation, with co-localization appearing as purple pixels. Analysis of staining patterns was performed by two investigators independently. The identity of the epitope being visualized was known to the investigators.

### Biochemical assays

Colo357 cells were grown as monolayers or spheroids. Measurements were repeated on six batches of lysates of monolayers or four batches of lysates of spheroids in a given size category. At the end of the incubation period, three aliquots of media and an aliquot of cell pellet (spun down at 1000 r.p.m. for 5 min to remove medium) were collected and cooled on ice to slow metabolism and membrane transport. In the case of spheroids, a small sample was extracted for microscopy to measure radius. Cells were lysed with 400 μl water (containing protease inhibitors) and two freeze-thaw cycles, and centrifuged (15 000 × *g* for 20 min at 4 °C) to remove cellular debris. [Lactate] and [glucose] were measured using an ABX Pentra 400 (Horiba, Northampton, UK) with the LDH and glucose PAP CP cassettes, respectively; osmolarity was measured by a Roebling freezing point osmometer (CamLab, Cambridge, UK); protein concentration was measured by standard BCA assay. Extracellular concentrations ([lactate]_e_, [glucose]_e_ and osmolarity) were measured in the aliquots of media. Intracellular concentrations were determined from lysates after correcting for the residual volume of medium that is inadvertently included with cells during collection. The lysate is a mixture of intracellular and residual medium volume fractions (*v*_i_, *v*_e_) and plus volume fraction of added water (*v*_w_=1−*v*_i_−*v*_e_). Assuming osmotic equilibrium between cells and media in culture, the sum of *v*_i_ and *v*_e_ is estimated from the lysate-to-medium osmolarity ratio (Osm_l_/Osm_e_). Lactate detected in lysates is from the intracellular fraction and residual medium. In contrast, lysate glucose is mainly from the residual medium because the majority of intracellular glucose is phosphorylated, and therefore not detected by the assays. Thus, glucose can be used as a marker of extracellular volume (*v*_e_). The following equations relate intracellular (i), extracellular (e) and lysate (l) concentrations:


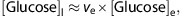



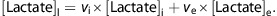


Cell-derived lactate in the lysate (mmol lactate_i_/L lysate) is therefore equal to


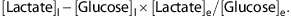


This result was normalized to the concentration of soluble protein in the lysate (units: mmol lactate_i_/g protein); to convert this into units of mmol lactate_i_/L cell (that is, mm), the result was multiplied by ρ: the mass of soluble protein in a unit of intracellular fluid (g protein/L cell). *ρ* was determined to be 13.21±1.78 g/l from the quotient of protein concentration (g protein/L lysate) and *v*_i_ (L cell/L lysate), calculated as:


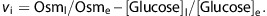


## Figures and Tables

**Figure 1:**
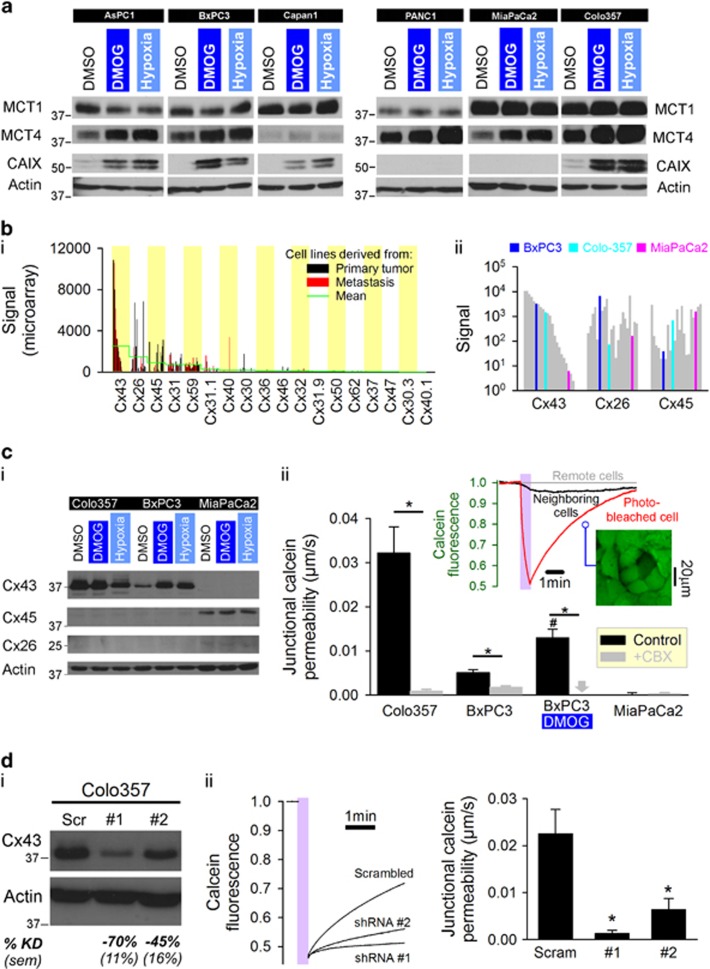
Gap junctional connectivity in PDAC cells. (**a**) Western blot (one of three repeats) for MCT1 and MCT4; CAIX induction is used as a marker of hypoxic signalling. (**b**(i)) Microarray data (BioGPS dataset E-GEOD-21654) for expression of connexin-coding genes in PDAC cell lines (see Supplementary Information for list; note that Cx23, Cx25 and Cx30.2 were not determined). (ii) Expression re-plotted on logarithmic scale, highlighting data for BxPC3, Colo357 and MiaPaCa2 cells. (**c**,i) Western blot (one of four repeats) for Cx43, Cx45 and Cx26 under normoxic conditions, after treatment with 1 mm dimethyloxalylglycine (DMOG), and incubation in 2% O_2_ (hypoxia). (ii) Fluorescence recovery after photobleaching (FRAP) protocol for measuring junctional calcein permeability. Specimen trace for Colo357 monolayer. Carbenoxolone (CBX; 100 μm) inhibited coupling. Mean±s.e.m. of 15 Colo357, 15 BxPC3 and 10 MiaPaCa2 cell clusters. Unpaired *t*-test *P<0.01 (control vs CBX), ^#^*P*<0.01 (DMOG vs normoxia). (**d**(i)) shRNA knockdown of Cx43 (*GJA1* gene) in Colo357 (lentiviral delivery) reduces Cx43 expression; two constructs (of four tested) with best knockdown efficiency are shown. Knockdown efficiency (% KD) determined densitometrically from the change in Cx43/actin ratio from three blots (mean±s.e.m.). (ii) Cx43 knockdown reduces cell-to-cell coupling assayed by FRAP. Note that enhanced green fluorescent protein (eGFP) signal associated with lentivirally-infected cells, is negligible (<10%) compared with calcein fluorescence and does not contribute to fluorescence recovery. Specimen time courses shown; histogram shows mean±s.e.m. (*n*=11, 8, 9); unpaired *t*-test *P<0.01.

**Figure 2:**
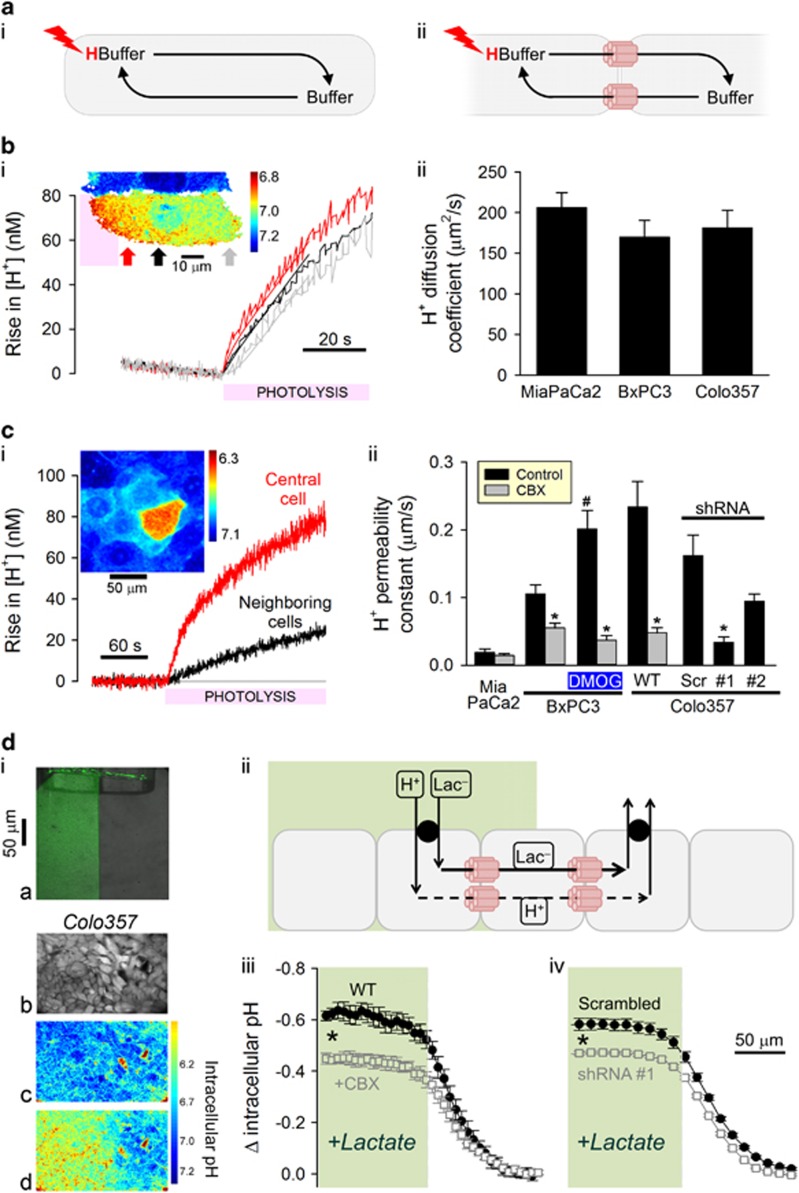
Junctional transmission of lactate and H^+^ ions. (**a**(i)) A source of H^+^ ions drives passive exchange of protonated and unprotonated buffers. Cytoplasmic H^+^ diffusivity probes buffer mobility in a cell. (ii) Cell-to-cell H^+^ permeability probes buffer transmission through gap junctions. (**b**(i)) Measuring H^+^ diffusion in a Colo357 cell, superfused in Hepes-buffered solution containing 1 mm NVA, the caged H^+^-compound. H^+^-uncaging (shaded region) produces a H^+^ microdomain that spreads diffusively in cytoplasm. Time courses of [H^+^] in three regions of interest (indicated by arrows) best-fitted with diffusion equation. (ii) Mean±s.e.m. (*N*=12, 15, 10). (**c**(i)) Measuring junctional H^+^ permeability between Colo357 cells. H^+^ ions were uncaged from 0.5 mm NVA in a central cell and time courses of [H^+^] in the central and neighboring cells derive junctional H^+^ permeability. (ii) Mean±s.e.m. (*N*=10, 11, 12, 10, 14, 12, 18, 10, 12, 14, 10). Experiments repeated in presence of carbenoxolone (CBX; 100 μm) and on *GJA1* knockdown cells. Unpaired *t*-test *P<0.01 (effect of CBX), ^#^*P*<0.02 (effect of DMOG). (**d**(i-a)) Regional exposure of a monolayer to 40 mm lactate-containing Hepes-buffered solution using a double-barrelled micropipette. For illustrative purposes, one microstream was labelled with 30 μm fluorescein; (b) fluorescence of cSNARF1-loaded monolayer; (c) pH_i_ before and (d) during regional exposure to lactate. (ii) Rapid junctional lactate transmission dissipates syncytial [lactate]_i_ gradient and permits further MCT-dependent H^+^-lactate entry into cells under the lactate-containing microstream (shaded green). (iii) Steady-state intracellular pH across monolayer of wild-type Colo357 cells measured after 2 min of regional exposure to lactate, minus intracellular pH under resting conditions. Experiment repeated in presence of CBX (100 μm). Mean±s.e.m. (*N*=7 WT, 10 CBX). (iv) Measurements on Colo357 cells transduced with shRNA #1 (knockdown) or scrambled construct. Mean±s.e.m. (*N*=5 WT, 5 CBX). ANOVA *P<0.05 for effect of CBX and for the effect of knockdown.

**Figure 3:**
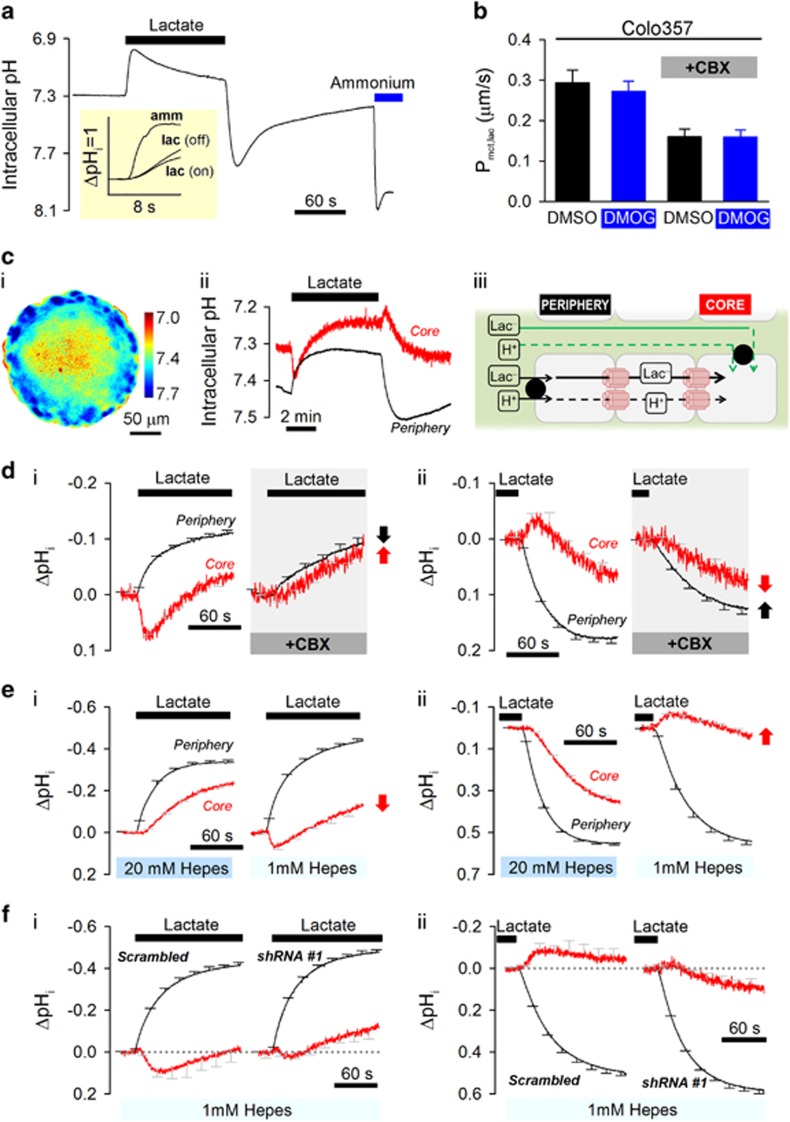
Intracellular lactate diffuses ahead of H^+^ ions in Colo357 spheroids. (**a**) pH_i_ response in cSNARF1-loaded Colo357 monolayer to 40 mm lactate-containing solution (probes MCT activity) followed by 30 mm ammonium-containing solution (tests speed of solution change). Inset: lactate-evoked pH_i_ changes are not rate-limited by solution change. (**b**) Membrane lactate permeability (*P*_mct,lac_) calculated from MCT activity. Gap junctional blocker CBX (100 μm) had partial inhibitory effect on *P*_mct,lac_. Mean±s.e.m. (*N*=20, 19, 20, 19). (**c**,i) pH_i_ map through equatorial plane of cSNARF1-loaded Colo357 spheroid (radius 110 μm). (ii) pH_i_ dynamics in peripheral layer (11 μm-thick rim; black time course) and core (radius 11 μm; red time course). (iii) Two routes of lactate entry into cells at the spheroid core. (**d**) pH_i_ response, in presence of CO_2_/HCO_3_^−^, to (i) adding and (ii) removing lactate. Biphasic response at core is blocked by CBX (100 μm; paired experiment). Mean±s.e.m. (*N*=8). (**e**) pH_i_ response, in absence of CO_2_/HCO_3_^−^, to (i) adding and (ii) removing lactate; extracellular buffering was provided by 20 mm or 1 mm Hepes (paired experiments). Mean±s.e.m (*N*=7). MCT activity at the core is rate-limited by slow H^+^ supply attained under a low (1 mM) [Hepes] buffering regime; this exposes a higher junctional component of lactate handling at the spheroid core. (**f**) Measurements in 1 mm Hepes performed on size matched spheroids grown from Colo357 cells transduced with shRNA #1 or scrambled construct. Unpaired measurements. Radius: 125.4±2.3 μm, 127.7±2.4 μm. Mean±s.e.m (*N*=5, 4).

**Figure 4:**
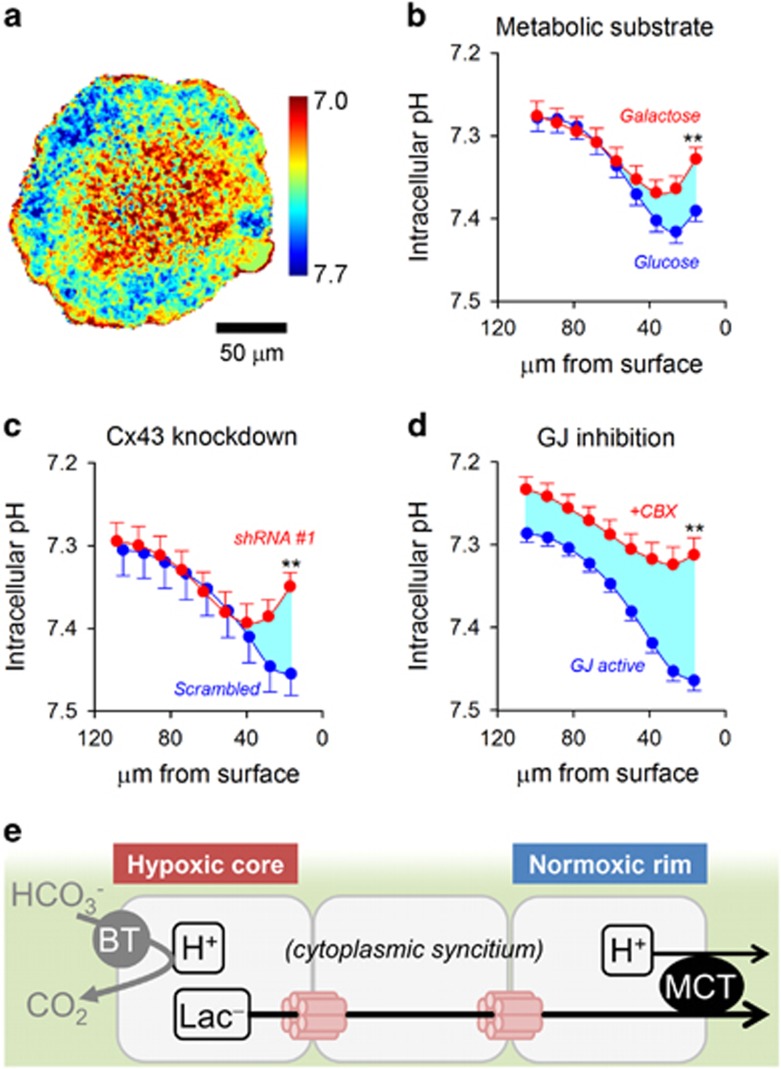
Junctional venting of lactate from the hypoxic core alkalinizes cells at the rim of Colo357 spheroids. (**a**) Steady-state pH_i_ across the equatorial plane of a Colo357 spheroid. (**b**) Radial pH_i_ gradient in Colo357 spheroids showing alkalinization at rim. Replacing glucose with galactose reduces degree of peripheral alkalinization. Unpaired *t*-test with Bonferroni correction, **P<0.01. Mean±s.e.m. (*N*=12 glucose, 8 galactose). (**c**) Peripheral alkalinization is smaller in Cx43-knockdown Colo357 spheroids (shRNA #1; Figure 1d) compared to scrambled controls. Mean±s.e.m. (*N*=14 shRNA #1, 12 scrambled). (**d**) CBX reduces peripheral alkalinization; the modest acidification at the core likely relates to the partial inhibitory off-target effect on MCT. Mean±s.e.m. (*N*=11 control, 8 CBX). (**e**) Mechanism by which junctional lactate transport alkalinizes recipient cells at the spheroid rim. Lactate and H^+^ ions are co-produced by hypoxic metabolism. HCO_3_^−^ (for example, taken-up by a bicarbonate transporter, BT) titrates cytoplasmic H^+^ ions in hypoxic cells.

**Figure 5:**
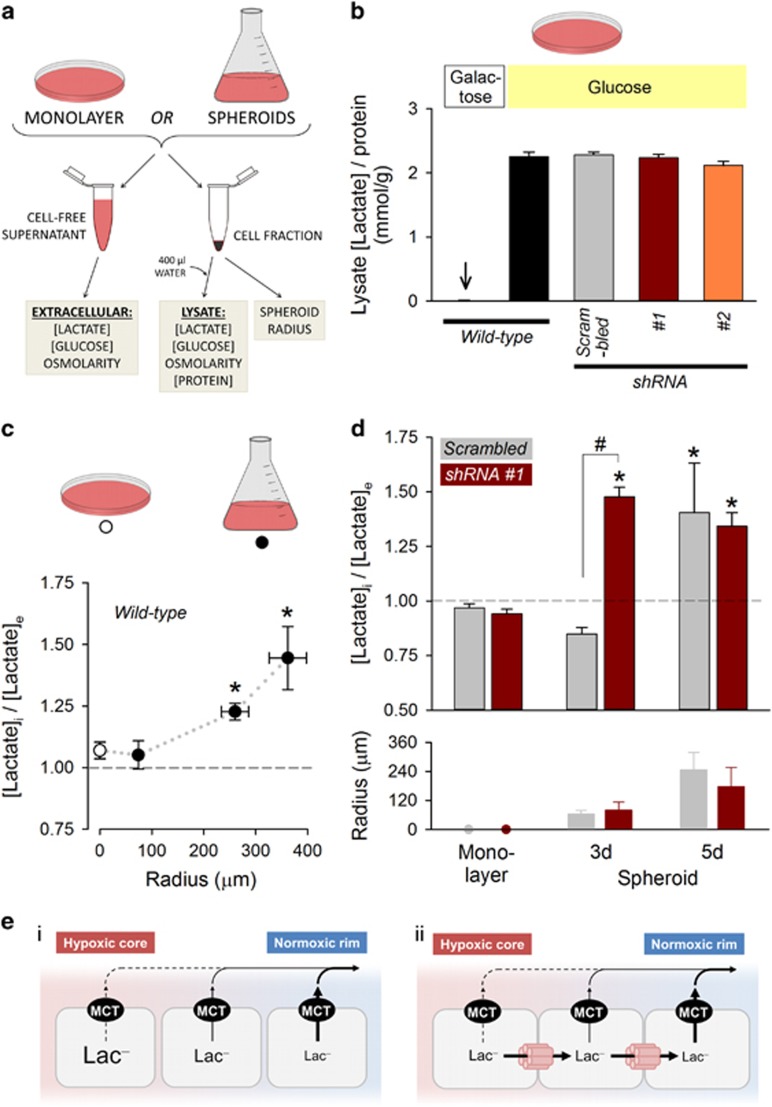
Intracellular lactate retention measured by biochemical assays. (**a**) Collection protocol: Colo357 cells were grown as monolayers for 96 h or as spheroids for 36–120 h. Between 30 and 100 spheroids were collected for measuring radius. Aliquots of medium and of cells were collected for biochemical assays; cells were lysed in 400 μl water and centrifuged to remove debris. (**b**) Lysates from monolayers grown in glucose- or galactose-containing medium were assayed for [lactate]/protein ratio. Lactate is produced only in the presence of glucose, and to comparable levels in wild-type and shRNA-transduced Colo357 cells (time-matched incubations). Mean±s.e.m. (six lysates each). (**c**) [lactate]_i_/[lactate]_e_ ratio (a measure of intracellular lactate retention) in wild-type monolayers (six lysates) and spheroids as a function of radius (four batches each containing >1000 spheroids). Mean±s.e.m. Unpaired *t*-test: * denotes significant increase in lactate retention relative to monolayer (*P*<0.05). (**d**) Upper panel: intracellular lactate retention in Colo357 cells infected with scrambled construct or shRNA #1, grown as monolayers (six lysates each) or as spheroids for 3 or 5 days. Mean±s.e.m. (four batches each containing >1000 spheroids). Unpaired *t*-test: * denotes significant increase in lactate retention relative to monolayer (*P*<0.05). ^#^ denotes significant difference between scrambled and shRNA #1 for duration-matched experiments (*P*<0.01). Lower panel: radius of spheroid, Mean±s.d. (**e**) Schematic diagram illustrating the routes for venting lactate. (i) In the absence of junctional coupling, all lactate traffic is through MCTs. Efflux in hypoxic regions with poor diffusive coupling is curtailed by an unfavourable transmembrane gradient; this leads to intracellular lactate retention. (ii) With junctional coupling, lactate can dissipate between cells and access normoxic regions that have a more favourable transmembrane gradient for MCT-facilitated off-loading; this reduces intracellular lactate retention.

**Figure 6:**
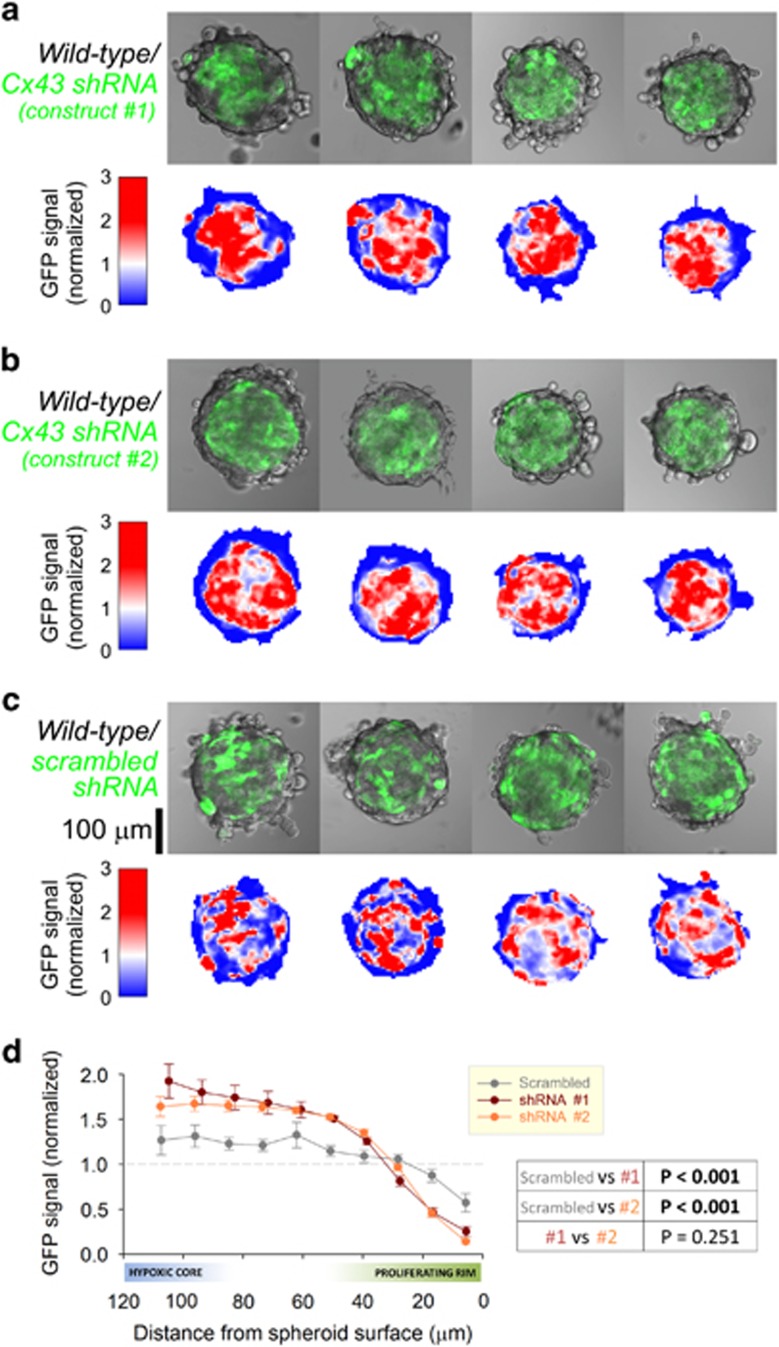
Spheroid co-cultures of Colo357 cells with different levels of Cx43. (**a**) Colo357 spheroid co-culture (1:1) of wild-type (non-fluorescent) cells mixed with eGFP-fluorescent *GJA1*-knockdown cells (shRNA construct #1). Upper panels: transmission image superimposed with fluorescence measured confocally across equatorial plane (pinhole=3 μm). Lower panels: fluorescence maps normalized to mean signal (blue pixels indicate below-average fluorescence; red pixels indicate above-average fluorescence), showing segregation by level of Cx43. Cx43-positive wild-type cells are more abundant in the peripheral rim. (**b**) Experiment repeated with the same imaging settings with shRNA construct #2, confirming the result with construct #1. (**c**) Experiment repeated with the same imaging settings with scrambled construct. Scrambled and wild-type cells mixed randomly, without clear radial segregation. (**d**) Analysis of eGFP fluorescence, normalised to spheroid-wide signal, as a function of radial depth: cells with high Cx43 levels segregate preferentially in the proliferating rim. Mean±s.e.m. (*N*=11 scrambled, 13 shRNA #1, 22 shRNA #2). Table shows results of ANOVA.

**Figure 7:**
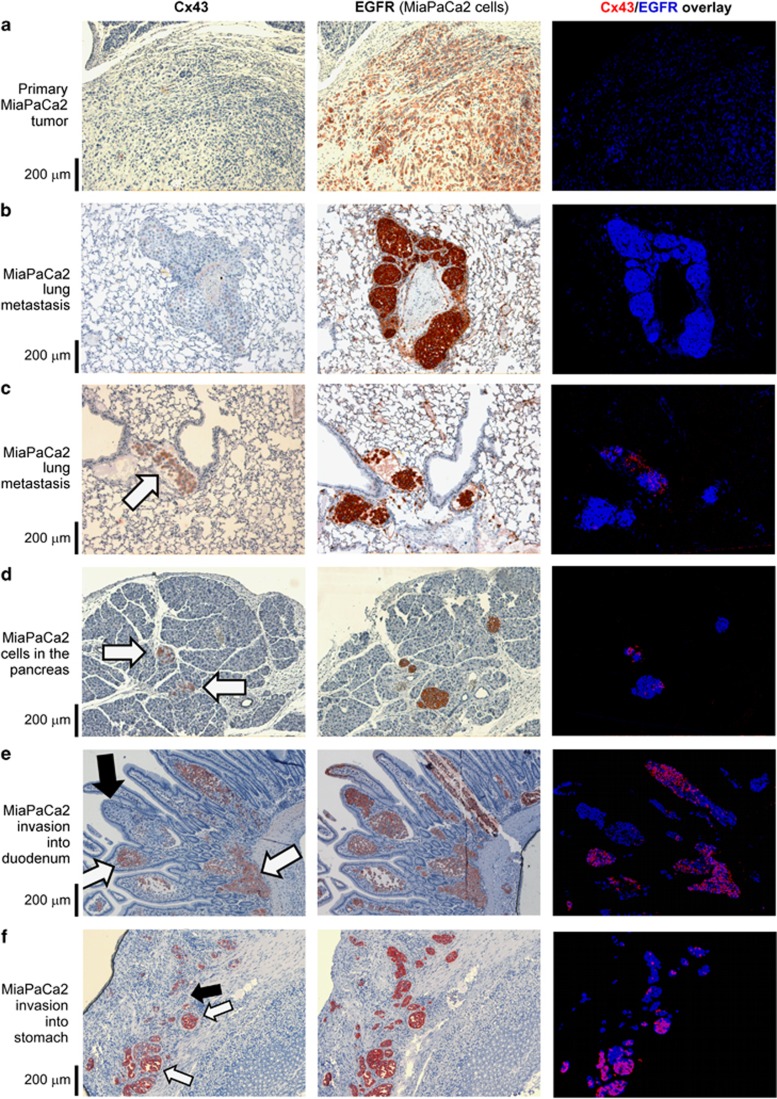
Cx43 immunoreactivity in MiaPaCa2 cells studied in orthotopic xenograft mouse models. Cx43 staining (left column) in sections of the primary tumour, invasion into adjacent tissues and sites of metastasis. Staining for human EGFR (middle column) identifies MiaPaCa2 cells. Overlay of Cx43 and EGFR immunoreactivity analysed by image-pair registration and colour filtering to identify staining (right column). (**a**) Cancer cells in the primary tumour remained Cx43 negative (overlay is blue, indicating EGFR signal only). (**b**) Example of a lung metastasis with absent Cx43 staining and (**c**) with more prominent Cx43 staining that co-localizes with an EGFR-positive cluster. (**d**) Clusters of Cx43-positive MiaPaCa2 cells (white arrows) within the host pancreas that co-localize with EGFR staining. MiaPaCa2 cell invasion into (**e**) mucosa of duodenum and (**f**) stomach muscularis, showing stronger and more uniform Cx43 staining (white arrows), but also a number of Cx43-negative clusters (black arrows). Clusters that are positive for both Cx43 and EGFR are indicated by purple colour on the overlay.

**Figure 8:**
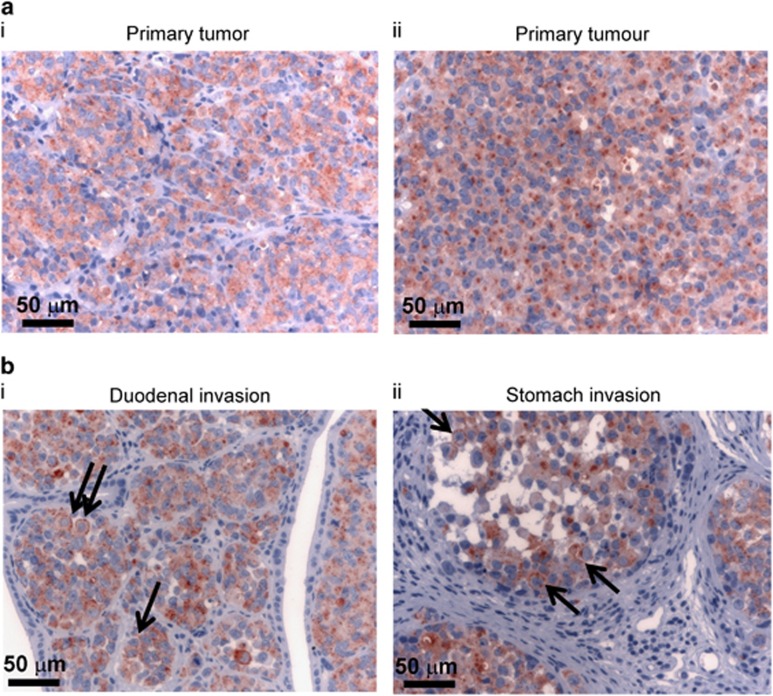
Distribution of LAMP2 in primary and invading MiaPaCa2 cells *in vivo.* (**a**) Diffuse LAMP2 staining in MiaPaCa2 cytoplasm but not at cell margins in sections of primary tumour. (**b**) In a subpopulation of MiaPaCa2 cells invading (i) duodenal mucosa and (ii) stomach muscularis, LAMP2 staining appeared more concentrated at cell margins, a marker of chronic extracellular acidosis.
