# Supplementary material for: Connexin-43 channels are a pathway for discharging lactate from glycolytic pancreatic ductal adenocarcinoma cells
Source: Oncogene. 2017 Apr 3;36(32):4538–50. doi: 10.1038/onc.2017.71 (PMC5507299; doi:10.1038/onc.2017.71)
Supplement: Supplementary Figures [file onc201771x1.pdf]

SUPPLEMENTARY FIGURES TO

Connexin-43 channels are a pathway for discharging lactate from glycolytic pancreatic ductal adenocarcinoma cells

TH Dovmark, M Saccomano, A Hulikova, F Alves and P Swietach

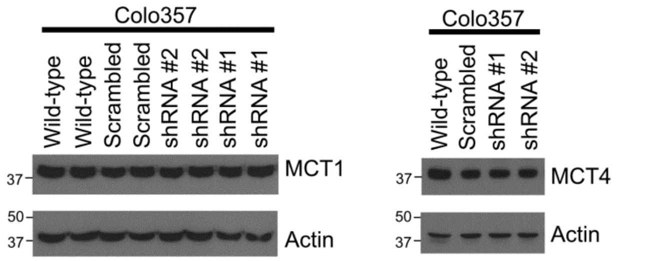

Figure S1. *MCT1/4 expression is unchanged by Cx43 knockdown.* Immunoblot showing that Colo357 cells infected with scrambled construct, shRNA #1 or shRNA #2 express MCT isoforms at comparable levels to wild-type cells.

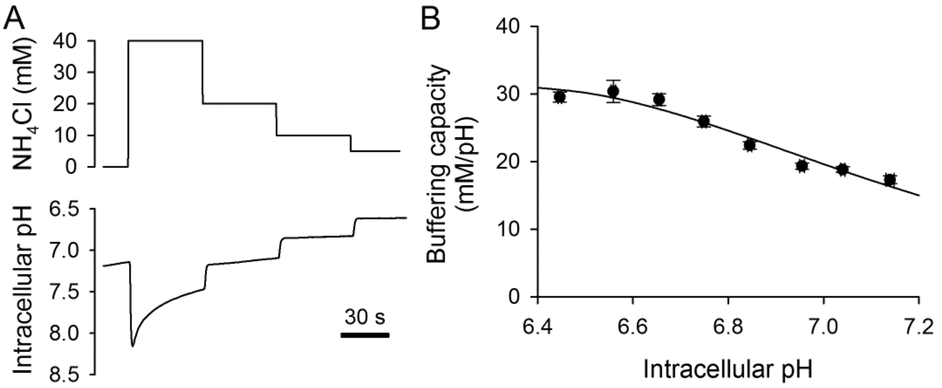

Figure S2. *Intrinsic buffering capacity.* (A) Colo357 monolayers, superfused in Hepes-buffered (CO_2/HCO_3^--free) solutions containing a range of [NH_4Cl] (iso-osmotically replacing [NaCl]), according to the protocol indicated in the upper panel. Measurements of intracellular pH show step-wise changes, coinciding with NH_4Cl removal; the amplitude of these is inversely related to buffering capacity. (B) Intrinsic buffering capacity as a function of intracellular pH, together with best fit curve. Each data point is mean±SEM from 20-35 cells.

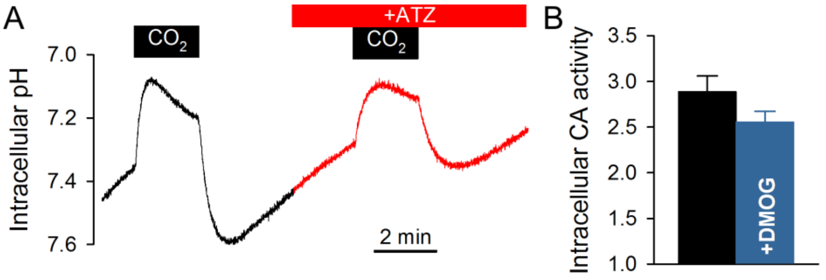

Figure S3. *Intracellular carbonic anhydrase activity.* (A) cSNARF1-loaded Colo357 monolayers under superfusion. Switching from Hepes-buffered to 5% CO_2/22 mM HCO_3^--buffered solution evokes CO_2 entry into cells and cytoplasmic CO_2 hydration, reported as a change in pH. The rate of the measured pH change depends on intracellular carbonic anhydrase activity. The uncatalysed hydration rate can be measured by repeating the protocol in the presence of acetazolamide (ATZ; 100 μM), a broad-spectrum carbonic anhydrase inhibitor. (B) Intracellular carbonic anhydrase activity in normoxic Colo357 cells and in cells with hypoxic signalling pathways stabilized with prior 48 hr incubation with 1 mM DMOG. CA activity is quantified as the measured CO_2 hydration rate constant divided by the uncatalysed rate constant; the result is dimensionless (a ratio of two first-order rate constants). Mean±SEM from 50 and 40 cells, respectively.

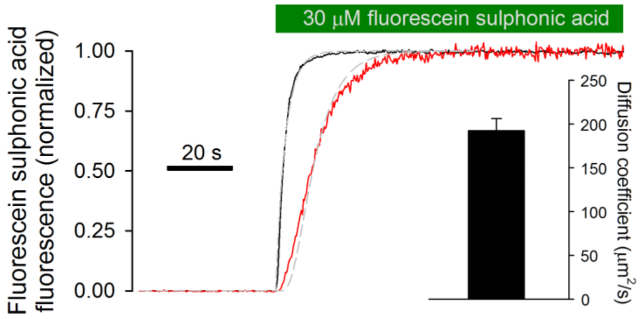

Figure S4. *Diffusive tortuosity in the extracellular compartment of Colo357 spheroids.* Colo357 spheroids (radius 110±5 μm) were superfused with Hepes-buffered solution at 37°C. At the point indicated (green bar), the superfusate was switched to one containing 30 μM fluorescein sulphonic acid, a membrane-impermeable fluorescent dye. Fluorescence was imaged across the equatorial plane of the spheroid. Black trace shows time course in the spheroid rim (layer of width=11 μm); red trace shows time course in spheroid core (radius=11 μm). Grey broken lines are model best-fit, as described in Hulikova & Swietach, 2015. The best-fit diffusion coefficient was determined from 9 spheroids; it was reduced by 70% relative to the diffusivity of fluorescein sulphonic acid in water at 37°C.

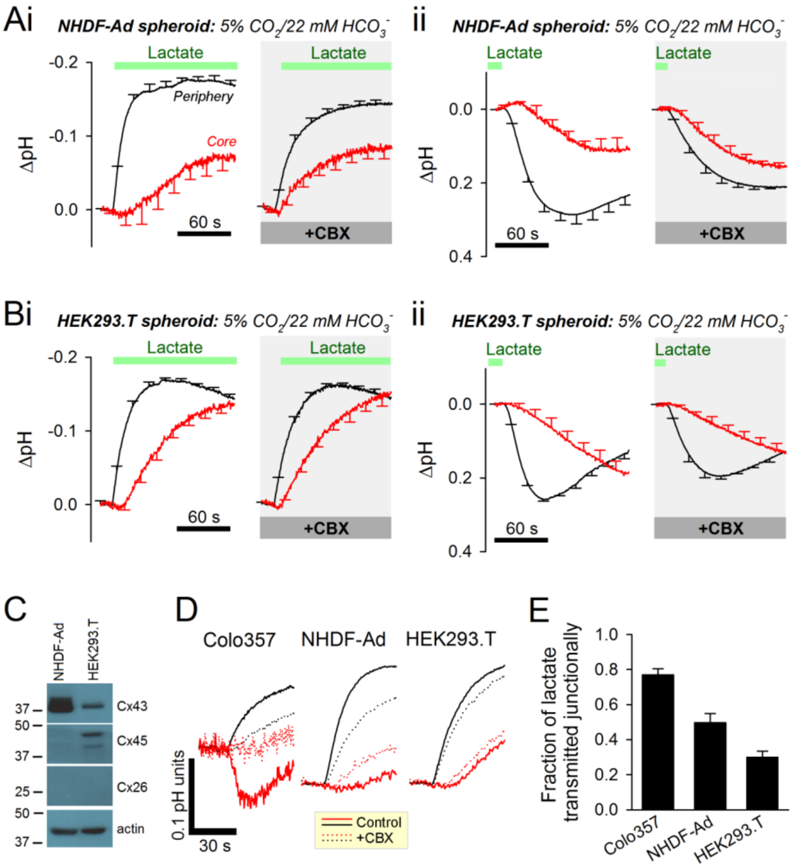

Figure S5. *Evidence for junctional lactate traffic in non-PDAC spheroids.* (A) NHDF-Ad (adult human fibroblast) spheroids (radius 101±2 μm) loaded with cSNARF1 and imaged across the equatorial plane for pH_i. Time courses show change in pH_i in the core *(red)* and rim *(black)*. Superfusion with 5% CO_2/22 mM HCO_3^--buffered solution (6 spheroids). Experiments also performed in the presence of 100 μM CBX to block gap junctions (7 spheroids). *(i)* pH_i response to superfusion with 40 mM lactate-containing solution and *(ii)* pH_i response to subsequent withdrawal of lactate. The pH_i time courses at the core and periphery appeared closer together under gap junctional inhibition. (B) Experiments repeated according to the above protocol on HEK293.T (human embryonic kidney cells) spheroids (radius 93±2 μm). (C) Immunoblot for Cx43, Cx45 and Cx26 expression in NHDF-Ad and HEK293.T cells. (D) pH_i time courses from Fig 3 (Colo357), S5A (NHDF-Ad) and S5B (HEK293.T), showing a range of responses. Colo357 spheroids demonstrated the greatest contribution from junctional lactate transmission, observed as a large alkalinising pH_i transient; the response was smaller in NHDF-Ad spheroids and weakest in HEK293T spheroids. These measurements correlated with Cx43 expression. (E) Fraction of lactate that is transmitted junctionally (see text).

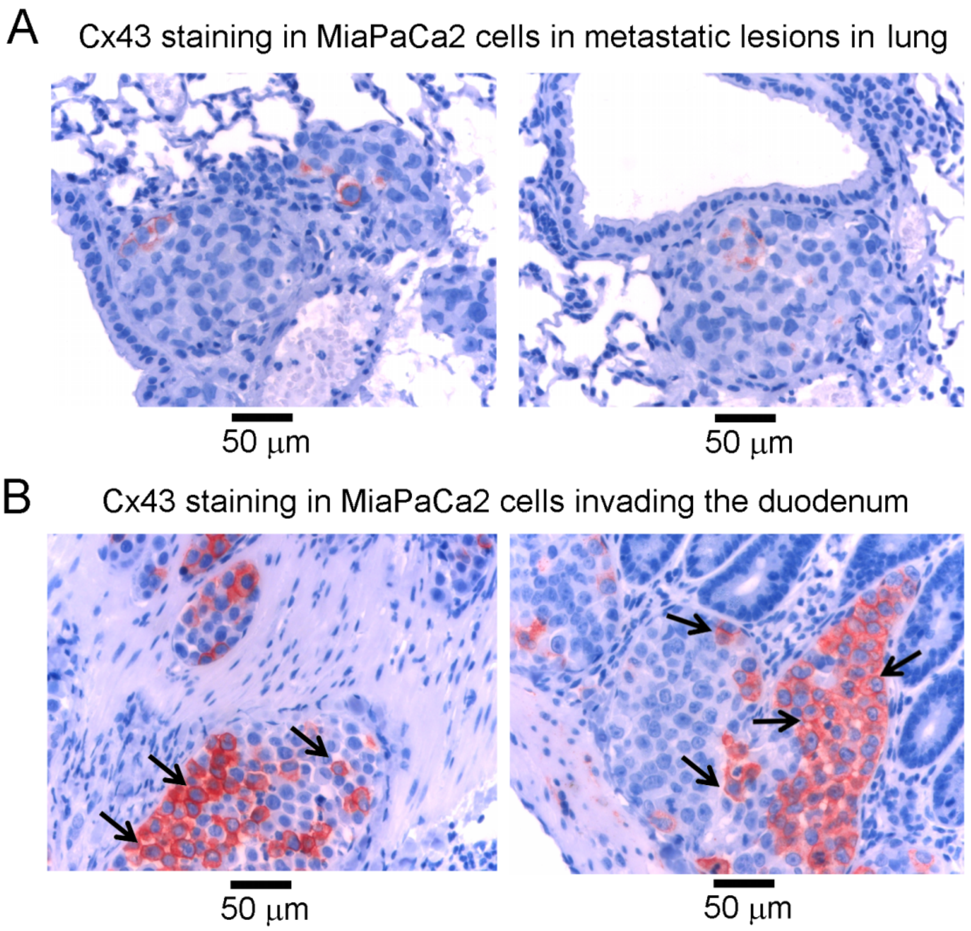

Figure S6. *Evidence for Cx43 staining at the membrane.* (A) Examples of two areas of lung (from different animals) with metastatic lesions containing MiaPaCa2 cells with sparse Cx43 staining. (B) Examples of two areas of duodenum (from different animals) containing clusters of invading MiaPaCa2 cells with strong Cx43 staining. Staining at cell-to-cell margins indicates surface membrane targeting, where Cx43 channels can form cell-to-cell conduits.

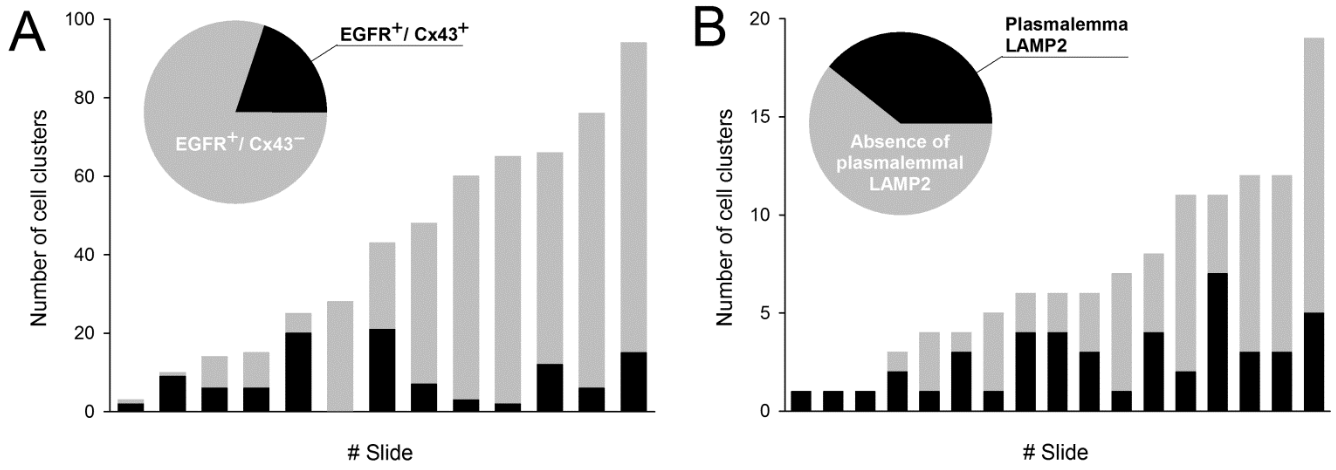

Figure S7. *Analysis of staining patterns in MiaPaCa2 cell clusters invading the gut or pancreas.* (A) Analysis performed on 13 sections of gut or pancreas from four different mice with orthotopic MiaPaCa2 xenografts. MiaPaCa2 cells were identified by positive EGFR staining. Out of a total of 547 clusters, 20% also showed strong Cx43 staining. (B) Analysis performed on 17 sections of gut or pancreas from four different mice with orthotopic MiaPaCa2 xenografts. MiaPaCa2 clusters were analysed for subcellular the pattern of LAMP2 staining. Out of 117 clusters, 39% included cells with LAMP2 staining near/at the plasmalemma, a marker of an acidotic niche.

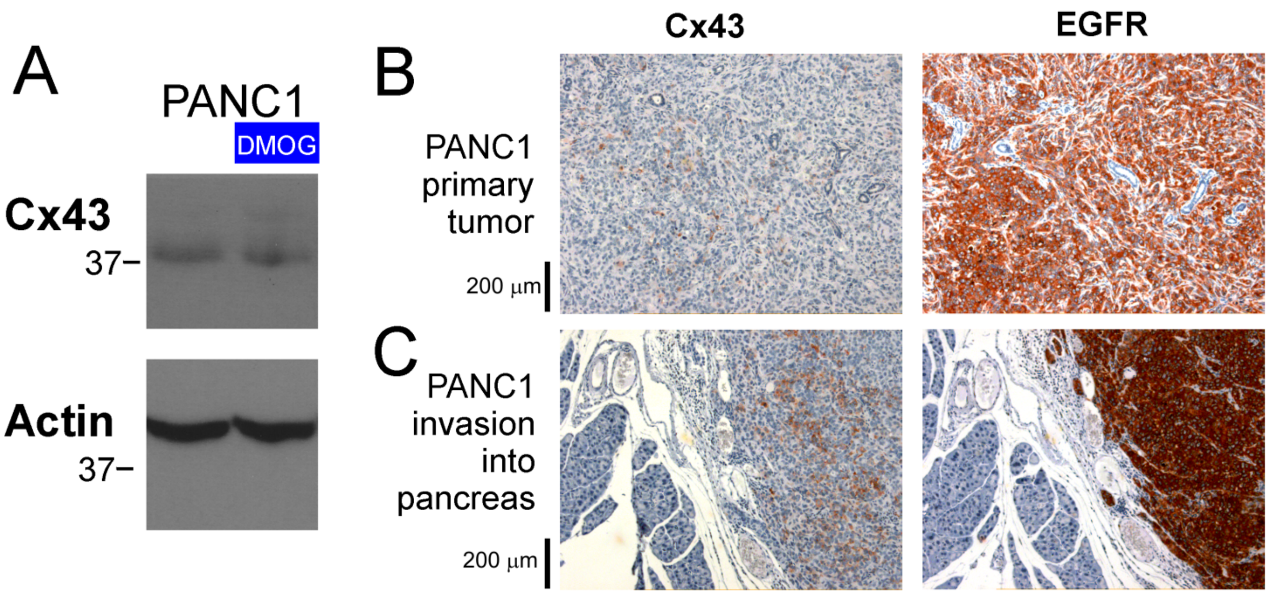

Figure S8. *Cx43 expression in PANC1 cancer cells in culture and* in vivo. (A) Low Cx43 expression in PANC1 cells cultured *in vitro* under normoxic conditions or in the presence of 1 mM DMOG (to stabilize hypoxic signalling). (B) Sparse pattern of Cx43 staining in regions of the primary tumour of PANC1 orthotopic xenograft mouse models. PANC1 cells are identified by positive staining for human EGFR. (C) Cx43 staining is increased in areas of invasion into the pancreas.
